# Prevalence and Factors Related to High Risk of Multiple Chemical Sensitivity among Japanese High School Students

**DOI:** 10.3390/ijerph21070934

**Published:** 2024-07-17

**Authors:** Tamami Suzuki, Yoshiko Bai, Yuko Ohno

**Affiliations:** 1 Graduate School of Nursing, Dokkyo Medical University, 880 Kitakobayashi, Mibu 321-0293, Japan; 2 Institutional Research Center, Dokkyo Medical University, 880 Kitakobayashi, Mibu 321-0293, Japan; baiz@dokkyomed.ac.jp; 3 Graduate School of Engineering, Osaka University, 1-1 Yamadaoka, Suita 565-0871, Japan; ohno@sahs.med.osaka-u.ac.jp

**Keywords:** multiple chemical sensitivity, high school students, QEESI, lifestyle behaviors, allergies

## Abstract

Multiple chemical sensitivity (MCS) onset in minors can greatly impact learning and future employment. This study investigated the prevalence of MCS and related factors in high school students to determine whether it was the same as in adults. A comprehensive survey was conducted on 80 high schools in Gunma Prefecture, Japan. The survey incorporated the Quick Environmental Exposure and Sensitivity Inventory, as well as items related to allergies, the living environment, and lifestyle. Of the 4630 students analyzed, according to Hojo’s cut-off value, 9.0% were classified as high-risk for MCS and 77.9% reported some allergy-like symptoms. Significant factors associated with elevated MCS risk included female sex, having various allergic conditions, having experienced living in a new home or home renovations or extensions, proximity to environmental stressors (freeways, national highway, factories, rubbish dumps, or sources of offensive odors), insufficient physical activity (exercising less than once a week outside of physical education classes), having cold hands and feet, being fatigued, having a bedtime earlier than 11 p.m., and having moderate–frequent subjective stress. Overall, 9.0% of high school students in Japan are at high risk for MCS. Enhancing awareness of MCS-like symptoms and addressing allergies, living environments, and lifestyle habits may mitigate these symptoms.

## 1. Introduction

Multiple chemical sensitivity (MCS), also known as environmental hypersensitivity, idiopathic environmental intolerance, and toxicant-induced tolerance loss, is a condition caused by prolonged exposure to small amounts of chemicals that are not normally considered harmful [1,2,3,4,5,6]. For example, fabric softeners and new building materials are not typically thought to have any effect on the human body. However, individuals with MCS can have severe reactions to these substances, causing a variety of symptoms that significantly impact their daily lives.

The symptoms of MCS include headaches, dizziness, difficulty breathing, palpitations, gastrointestinal symptoms, fatigue, depressive symptoms, and cognitive impairment [1,2,3,4]. In Japan, MCS was included in the 10th edition of the International Classification of Diseases in 2009; however, the etiology, diagnosis, and treatment of MCS remain controversial [1,5,7,8], and clear physiological diagnostic criteria have not been established. The 1999 consensus provides the most comprehensive case definition [1,7,9,10], and recommends the Environmental Exposure and Sensitivity Inventory (EESI) and its shortened version, the QEESI, for screening MCS [11].

Although there are few reports that calculate the prevalence of MCS based on clinical assessment or the 1999 consensus criteria, existing studies have provided some estimates. The estimated prevalence is 0.5–12.8%, based on medical diagnosis, or 0.9–33.0%, based on self-reported information [5]. These figures suggest that a significant number of individuals experience and are aware of MCS-like symptoms, despite some debate about the condition’s existence. Between 2002 and 2016, the number of individuals with MCS-like symptoms increased two- to three-fold in the United States [4]. Although the onset of MCS in minors can significantly impact educational outcomes and future employment, most research has predominantly focused on adults [12].

In Japan, the prevalence of MCS was 12.4%, based on a survey targeting first-year elementary school students to third-year junior high school students [12]. The issue of health problems caused by school facilities, including how to deal with children with chemical sensitivities, is known as the “sick school problem”, prompting cities and prefectures to create countermeasure manuals. However, there are few studies on the prevalence of chemical sensitivity in minors and factors related to its onset, and there have been no studies targeting high school students. High school students, with their still-developing bodies and prolonged exposure to communal environments like schools, may exhibit distinct prevalence rates and risk of MCS compared with adults; however, this potential difference remains unexplored due to a lack of research. In Japan, high school students are choosing their career paths; however, the prevalence of MCS, the degree of hypersensitivity to chemical substances, and related factors that may affect career choices and studies remain unclear.

Therefore, in this study, we used the globally recognized QEESI [13,14,15,16,17,18] to screen for chemical sensitivity among high school students. Our objectives were to calculate the proportion of high school students at high risk of MCS and examine the related factors. Our findings indicate that a significant proportion of high school students are at high risk of MCS, with several lifestyle and environmental factors associated with increased risk. These results highlight the importance of addressing chemical sensitivity in adolescent populations and suggest potential areas for intervention and further research.

## 2. Materials and Methods

### 2.1. Participants

This study surveyed high school students from Gunma Prefecture in Japan, covering 80 (68 public schools and 12 private schools) of the 87 high schools listed on the Gunma Prefectural Education Center website. Seven special high schools were excluded (correspondence high schools and residential boarding high schools).

### 2.2. Survey Method

To calculate the percentage of high school students who were highly sensitive to chemical substances, we used the QEESI [11], which has been used both in Japan [19,20,21,22,23,24] and internationally [16,17,25,26,27,28,29,30,31,32,33] as a screening tool for MCS. The QEESI was developed by Miller and Prihoda in the USA in 1999 for screening MCS patients and evaluating the effectiveness of treatment, and for epidemiological research on MCS using self-reporting questionnaires. Currently, it has been translated into 16 languages; in Japan, it was translated by Ishikawa and Miyata in 1998, and its reliability and validity were confirmed by Hojo et al. in 2003. The QEESI results are classified based on the cutoff value. Commonly used cutoff values in MCS research include those developed by Miller et al. [11], Hojo et al. [19], and Skovbjerg et al. [18]. For this study, we opted to use the cutoff value established by Hojo et al., which was specifically tailored for Japan.

We requested study cooperation from the 80 target schools by mail. Questionnaires were sent only to the high schools that consented to cooperate, and the survey was implemented after the aims and methods were explained to the students by teachers at each high school. The questionnaires were returned to the researchers after being collected at each high school. Data collection was conducted from July to October 2013.

### 2.3. Survey Content

The questionnaire, which included 100 questions, was designed to be answered in less than 15 min. It covered the following surveyed items:

(1) Attributes (e.g., sex, school year), diagnosis of sick house syndrome (SHS), and diagnosis of MCS.
(2) Allergies, including hay fever, atopic dermatitis, asthma, hives, and other allergies, assessing whether the respondent currently had symptoms or had symptoms in the past.
(3) Living environment (i.e., whether the respondent lived in a new building or a building undergoing renovations) and details about the environment surrounding the home.
(4) Lifestyle (including various aspects of daily habits and routine).
(5) QEESI [19] sections, specifically QEESI Q1 (Chemical Intolerance), Q3 (Symptom Severity), and Q5 (Impact on Life). Each section comprised 10 questions, scored on a 0–10 scale, resulting in a total score range of 0 to 100 points for Q1, Q3, and Q5.

### 2.4. Statistical Analysis

Hojo’s cut-off value [19] was used to identify high school students at high risk of MCS (hereinafter referred to as the MCS high-risk group) and participants with Q1, Q3, and Q5 scores ≥40, ≥20, and ≥10, respectively, were assigned to the “MCS high-risk group”.

SPSS version 21 (IBM Corps., Armonk, NY, USA) was used to compare the groups. Before selecting the appropriate statistical tests, we first assessed the normality of our data using the Shapiro–Wilk test. This step was crucial in determining whether to use parametric or non-parametric methods in our subsequent analyses. As a result, the Mann–Whitney U test was used for continuous data, and Pearson’s Chi-squared test was used for categorical data. In our cross-tabulation analyses, we applied a specific criterion for using Fisher’s exact test. This test was employed when there were cells with expected frequencies less than 1, or when expected frequencies less than 5 accounted for 20% or more of the total cells. This approach ensured accurate analysis in cases where the assumptions of the Chi-squared test might not have been met due to low expected cell counts. The crude odds ratio (COR), adjusted odds ratio (AOR), and 95% confidence interval (95% CI) were calculated. In the univariate analysis, items with *p* values < 0.25 were selected as independent variables, and a logistic regression analysis was conducted using the variable increase method (likelihood ratio). The level of significance was set at less than 5%. To evaluate multicollinearity, the variance inflation factor (VIF) was obtained. When the VIF was 3 or more, it was determined that there was collinearity between the independent variables, and the variable was subsequently excluded.

### 2.5. Ethical Considerations

Prior to the survey, the study was explained to the participants by their teachers at each high school, and an explanation and consent form were distributed. It was explained to participants that “participation/cooperation in the study is voluntary”, “consent to participate in the study is granted through submission of the questionnaire”, “the survey is anonymous”, and “the acquired data would be promptly digitized, strictly controlled by storage in a locked storage cabinet, and destroyed as soon as the survey results were compiled”. After the questionnaire was completed, the students placed their questionnaires into sealable individual envelopes, which were then sealed and placed into a collection bag.

This study was conducted in accordance with the principles outlined in the Declaration of Helsinki of 1975, revised in 2013, and approved by the Institutional Review Board of Gunma PAZ University (protocol code: 13–17; date of approval: 24 July 2013).

## 3. Results

### 3.1. Participant Characteristics

**Figure 1:**
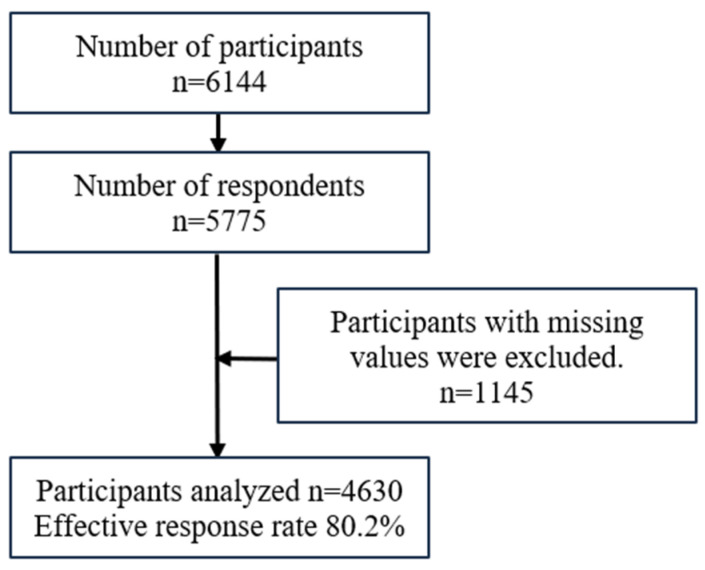
Flow chart of participant selection.

Twenty-one schools (26.3%) agreed to participate in this study. Questionnaires were distributed to 6144 students and responses were obtained from 5775 students (response rate, 94.0%). The data of 4630 students without missing data (effective response rate, 80.2%) were analyzed (Figure 1).

Of the 4630 students, 2413 were male (52.1%) and 2217 were female (47.9%); 2342 students were in their first year of high school (50.6%), 1639 in their second year (35.4%), and 649 in their third year (14.0%) (Table 1).

Using Hojo’s cut-off value [19], a total of 415 students (9.0%) were assigned to the MCS high-risk group. The remaining 4215 students (91.0%) were considered as the control group. Factors that affected the MCS high-risk group and the control group were then investigated.

### 3.2. Sex, Diagnosis, and Symptoms

The correlation between sex and the MCS high-risk group is shown in Table 2. The percentage of females in the control group versus the MCS high-risk group was 46.9% versus 57.8%, respectively (*p* < 0.001, COR: 1.55).

The correlations of the MCS high-risk group with diagnosis, subjective symptoms, and allergy-like symptoms are shown in Table 2. Among the entire cohort, 0.4% (n = 19) were diagnosed with SHS, and 0.5% (n = 24) were diagnosed with MCS. A significant correlation was found between “having an SHS diagnosis” and the MCS high-risk group (*p* = 0.023, COR: 3.67); however, no significant correlation was found between “having an MCS diagnosis” and being in the MCS high-risk group (*p* = 0.267).

Of the 4630 participants, 61.1% had hay fever, 30.9% had urticaria, 27.0% had mold/dust/mite allergies, 18.2% had atopic dermatitis, and 16.8% had asthma. Overall, 77.9% of the students experienced allergy-like symptoms. Students with allergies accounted for 77.5% of the control group and 82.4% of the MCS high-risk group, and allergies were significantly related to the MCS high-risk group (*p* = 0.022, COR: 1.36).

In addition, the MCS high-risk group was associated with atopic dermatitis (*p* = 0.009, COR: 1.38), mold/dust/mite allergies (*p* = 0.006, COR: 1.36), urticaria (*p* = 0.012, COR: 1.31), food allergies (*p* = 0.003, COR: 1.48), drug allergies (*p* = 0. 007, COR: 1.94), and metal allergies (*p* < 0.001, COR: 2.57). Metal allergies showed the highest COR.

### 3.3. Number of Allergy-like Symptoms

To examine whether the allergy-like symptoms were diverse, we counted the number of diseases with allergy-like symptoms, including hay fever, atopic dermatitis, asthma, mold/dust/mite allergies, urticaria, food allergies, drug allergies, and metal allergies. If all were applicable, this counted as eight allergy-like symptoms. We found that the MCS high-risk group tended to have a higher percentage of students with allergy-like symptoms than the control group (Table 3).

### 3.4. Residential Environment

The comparison of residential environmental factors between the MCS high-risk group and the control group is shown in Table 4. The following items were significantly correlated with the MCS high-risk group: the number of times the respondent had experienced moving into a new home; home renovations or extensions; and proximity to environmental stressors, including living close to a freeway, national highway, factory, rubbish dump, or a source of offensive odors.

### 3.5. Lifestyle Habits

The comparison of residential lifestyle between the MCS high-risk group and the control group is shown in Table 5. No statistically significant differences in residential lifestyle were found between the MCS high-risk group and the control group.

The comparison of lifestyle factors between the MCS high-risk group and the control group is shown in Table 6. The only statistically significant item was “Exercises more than once a week outside of physical education class”, which was significantly lower in the MCS high-risk group.

The comparison of lifestyle habits and physical/mental conditions between the MCS high-risk group and the control group is shown in Table 7. The MCS high-risk group reported significantly higher rates of “cold hands and feet”, “fatigue”, “subjective stress”, and the number of “yes” responses compared with the control group.

### 3.6. The Three QEESI Category Scores

Intergroup comparisons of the three QEESI category scores are shown in Table 8. These comparisons were made using the Mann–Whitney U test, due to the non-normal distribution of our data. When comparing the median values of the control and MCS high-risk groups across the three QEESI categories (Q1: chemical intolerance, Q3: symptom severity, and Q5: impact on life), all showed significantly higher values in the MCS high-risk group, as indicated by the Mann–Whitney U-test results.

### 3.7. Logistic Regression Analysis of Factors Related to the MCS High-Risk Group

**Figure 2:**
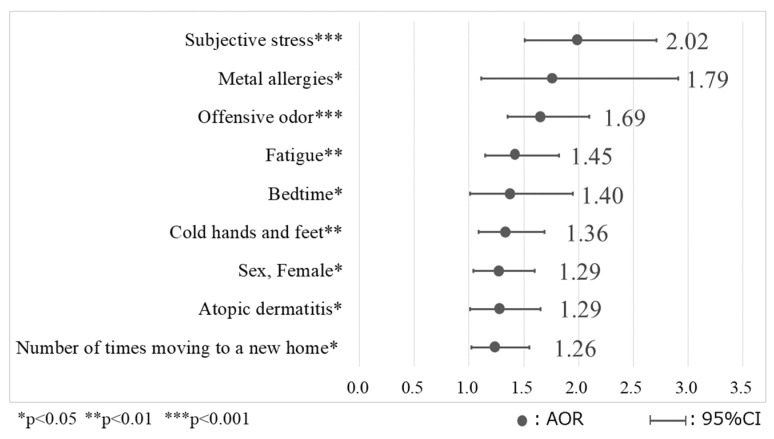
Factors related to the MCS high-risk group.

The results of the logistic regression analysis of factors related to the MCS high-risk group are shown in Table 9 and Figure 2. When the *p* value was set at ≤0.25, 32 variables were selected, and when the *p* value was set at ≤0.15, 25 variables were selected; both methods were stable with nine steps and nine similar variables.

The model included nine variables consistently showing significance among the total 57 variables included in the model: “female sex”, “having atopic dermatitis”, “having metal allergies”, “having cold hands and feet”, “being fatigued”, “moving into a new home one or more times”, “exposure to offensive odors”, “bedtime earlier than 11 p.m.”, and “subjective stress: moderate–frequent”. The results of the Chi-squared test for the model were significant (*p* < 0.001), with each variable individually showing significance as well (*p* < 0.05). The Hosmer–Lemeshow test was used to examine the predictive accuracy of the model and showed a high degree of predictive accuracy (*p* = 0.976); the discriminative predictive value was 91.0%.

## 4. Discussion

In this study, the prevalence of high sensitivity to chemicals (MCS high-risk group) in Japanese high school students was 9.0%, and the following factors were associated with a high risk of MCS among high school students: female sex, having multiple allergies, frequent relocation into a new home (once or more), having experienced home renovations or extensions, proximity to environmental stressors (living near a freeway, national highway, factory, rubbish dump, or source of offensive odors), not exercising once or more per week outside of physical education classes, having cold hands and feet, being fatigued, having a bedtime earlier than 11 p.m., and having moderate–frequent subjective stress.

### 4.1. Prevalence of MCS-like Symptoms

As mentioned above, the percentage of high school students who were classified as chemically sensitive (MCS high-risk group) according to the QEESI was 9.0%. Previous studies that have used the QEESI have estimated the prevalence to be 20.4% in the United States [15], 8.2% in Denmark [18], 14.4% in South Korea [17], and 7.5% in Japan [34]. However, in those studies, the target population comprised mainly adults and the cut-off values were different; therefore, the estimates cannot be simply compared. Furthermore, in surveys targeting minors, the rate was 15.6% in Sweden (13–19 years old) [35] and 12.4% (6–15 years old) in Japan [12]; however, the data were self-reported and the QEESI was not used. Therefore, a simple comparison is similarly difficult. However, the finding that 9.0% of high school students were in the high-risk group for MCS indicated that 9.0% of high school students exhibited symptoms resembling those associated with MCS, regardless of whether they recognized these symptoms as being related to MCS. It is believed that the awareness of MCS will be improved and the QEESI will be disseminated so that high school students can receive appropriate diagnosis and treatment.

The association between having been diagnosed with SHS and being in the MCS high-risk group was statistically significant; however, there was no association between having been diagnosed with MCS and being in the MCS-high-risk group. This association may not have been significant, due to there being a limited number of students diagnosed with MCS; thus, the results are difficult to interpret. In Japan, MCS is less well known than SHS. Furthermore, diagnostic criteria for MCS have not been established, and MCS presents with various symptoms, making it difficult to see a specialist. In addition, the QEESI scores may have been low because those with severe MCS-like symptoms may not have been able to attend school and take the survey; alternatively, they may have received treatment and reconsidered their lifestyles, leading to the disappearance of symptoms [13,36].

### 4.2. Allergy-like Symptoms and MCS

In this study, 77.9% of participants had some type of allergy-like symptoms (hay fever, atopic dermatitis, asthma, mold/dust/mite allergy, hives, food allergies, drug allergies, or metal allergies). As previous studies have not assessed the overall prevalence using identical survey items, we present our results by comparing each item with findings from related surveys. Specifically, the figure of 77.9% represents the proportion of individuals experiencing any of the eight specified allergy-like symptoms in our study. To contextualize these findings, we compared the incidence rates of each symptom with those reported in the existing literature. The results of this study are shown first and the results of previous studies are shown in parentheses: hay fever, 61.1% (48.1% [37]); atopic dermatitis, 18.2% (24.7% [38]); asthma, 16.8% (18.8% [39]); mold/dust/mite allergy, 2.7% (18.8% [40]); urticaria, 30.9% (8.8/20% [41,42]); food allergies, 13.8% (16.8% [43]); drug allergies, 2.7% (3.9% [44]); and metal allergies, 2.5% (3% [45]). While direct comparisons are complicated due to variations in factors such as prevalence rate, calculation methods, participant age, country of residence, and survey year, our study’s findings notably indicate relatively high rates of hay fever and urticaria. However, in a survey conducted in Tochigi Prefecture, a prefecture adjacent to Gunma Prefecture where this survey was conducted, 27.7% of elementary and junior high school students answered that they had no allergies [43], which is close to the 22.1% reported in this study. Therefore, it is not unreasonable to consider the reported 77.9% prevalence of allergy-like symptoms among high school students as credible, given the findings from these comparable studies.

Previous studies have reported that MCS is more common in individuals with allergy-like symptoms [21,28,32,34,46] and women [47], and the present study revealed similar results. The number of individuals with allergy-like symptoms is increasing yearly [40], and people with allergy-like symptoms are more likely to develop chemical sensitivity [20,28,29]. There is concern that the number of patients with MCS will increase with an increase in the number of individuals experiencing allergy-like symptoms.

Regarding the types of allergies, atopic dermatitis [32,34,46] and metal allergies [48], which were found to be statistically significant in previous studies, were also significantly associated with the MCS high-risk group in this study. However, regarding bronchial asthma [4,14,49,50], this study found no significant association in either the single or multivariate analyses.

Although the relationship between allergy-like symptoms and MCS requires further investigation, it is necessary to raise awareness of MCS so that people with allergy-like symptoms are aware of the onset of MCS symptoms.

### 4.3. Factors Related to the MCS High-Risk Group and Prospects for the Prevention of MCS Onset

In this study, we examined factors associated with a high risk of MCS, such as sex, presence of allergy-like symptoms, living environment, and lifestyle.

Regarding sex, the probability of being female in the MCS high-risk group was 1.55 times the probability of being female in the control group; this finding is similar to those of previous studies of adults [32,34,35,47]. In Japan, it is believed that one of the causes of MCS is sick building syndrome and that women are more likely to develop SHS/MCS than men because they spend more time exposed to radiation in homes that emit chemical substances. However, as the participants of this survey were high school students, it is unlikely that the amount of time spent at home differed between men and women, suggesting that there may have been another reason. In the future, we would like to consider the possibility that women have higher percentages of body fat than men and that chemicals are more likely to accumulate there.

Regarding the living environment, the finding that people who had experience of moving into a new house tended to be more likely to develop MCS was similar to findings in previous research [32]. When moving into a new house, renovating, expanding, or remodeling, it is important to select and use materials that emit the smallest possible amount of volatile chemicals and to take measures to ensure that volatile chemicals evaporate sufficiently. However, it is difficult for individuals to improve the environment around their homes, and relocating is not an easy option. Therefore, if there are expressways, national highways, railways, factories, or garbage disposal sites nearby, it is important to take measures as far as possible to prevent exposure to chemical substances [51].

It is understood that as symptoms of MCS increase in severity, sensitivity to odors increases as a protective response against potentially harmful substances [13,51]. This sensitivity also plays a crucial role in encouraging immediate departure from environments or objects emitting unfamiliar odors.

Regarding lifestyle habits, items such as “cold hands and feet”, “feeling of fatigue”, “subjective stress”, “bedtime earlier than 11 p.m.”, and “exercising less than once a week” were associated with the high-risk group for MCS. Regarding “cold hands and feet”, it is believed that “coldness” causes circulatory failure, which causes detoxification and chemical excretion functions to no longer work smoothly. Recently, “Onkatsu”, which refers to warming the body to improve blood circulation and ultimately enhance sensitivity to cold and various other ailments, has been attracting attention in Japan. Further research is expected to determine whether Onkatsu and improving coldness are effective ways to improve and prevent the onset of MCS.

Regarding bedtimes, a study of Japanese children aged 6–15 years stated that later bedtimes were associated with MCS [12]. However, in this study, earlier bedtimes were more common in the MCS high-risk group. It was difficult to compare the results directly because the ages of the survey participants were different. Therefore, further research is needed to investigate the relationship between bedtime and MCS; it is possible that MCS-like symptoms may lead to earlier bedtimes. Regardless, although the recommended bedtime for individuals in this study was unknown, there was an evident relationship between MCS-like symptoms and “bedtime”, “feelings of fatigue”, and “subjective stress”. Hence, stress management and lifestyle adjustments are important.

Regarding the habit of exercising more than once a week, sweating during exercise helps eliminate waste products, increases muscle mass, increases basal metabolism, and creates a constitution that makes it easier to eliminate chemicals. It is expected to be useful in improving and preventing the onset of MCS.

Previous studies have reported that MCS-like symptoms can be improved by improving the air quality in the living environment [13,34,51]. It is necessary to disseminate information about MCS so that people can take measures as far as possible to avoid exposure to chemical substances and incorporate coping methods, such as stress management, lifestyle adjustments, and exercise therapy, to improve and prevent the onset of MCS-like symptoms.

### 4.4. Strengths and Limitations

This was the first study to examine the prevalence of high-risk MCS and associated factors in high school students. The data obtained from the MCS survey of thousands of high school students are extremely valuable. In addition, with a response rate of 94.0%, the ratio of the MCS high-risk group to the control group within participating schools provides valuable insights for generalization and serves as valuable epidemiological data.

This study also had some limitations. First, this study was conducted in 2013; hence, the current situation may be different. Second, because this study was based on a self-administered questionnaire and was a cross-sectional study, it was not possible to clarify causal relationships, and we could only speculate about the associations. Third, different samples may have yielded different results because of the small number of high school students with MCS-like symptoms. Fourth, this study targeted high school students in Gunma Prefecture, which may have influenced the results of items related to regional characteristics, such as hay fever and the living environment.

## 5. Conclusions

Overall, the percentage of Japanese high school students who were sensitive to chemical substances (MCS high-risk group) was 9.0%, and 77.9% reported allergy-like symptoms. The following factors were related to a high risk of MCS in high school students: female sex, having various allergic conditions, relocating into a new home once or more, having experienced home renovations or extensions, living near a freeway/national highway/factory/rubbish dump/source of offensive odors, not exercising once or more per week outside of physical education classes, having cold hands and feet, being fatigued, having a bedtime earlier than 11 p.m., and moderate–frequent subjective stress. The results suggest that 9.0% of high school students in Japan are aware of MCS-like symptoms. Addressing allergies, the living environment, and lifestyle habits may lead to the improvement of MCS-like symptoms.

## Figures and Tables

**Table 1:** Characteristics of the participants.

Characteristics	Total Number n = 4630 (100%)		Control Group n = 4215 (91.0%)		High-Risk Group n = 415 (9.0%)		*p*-Value ^1^
	N	(%)	n	(%)	n	(%)	
**Sex**							<0.001
Male	2413	(52.1)	2238	(53.1)	175	(42.2)	
Female	2217	(47.9)	1977	(46.9)	240	(57.8)	
**Grade**							0.711
Sophomore	2342	(50.6)	2129	(50.5)	213	(51.3)	
Junior	1639	(35.4)	1499	(35.6)	140	(33.7)	
Senior	649	(14.0)	587	(13.9)	62	(14.9)	
**Course**							0.189
General	3013	(65.1)	2734	(64.9)	279	(67.2)	
Commercial	568	(12.3)	522	(12.4)	46	(11.1)	
Industrial	628	(13.6)	574	(13.6)	54	(13.0)	
Another	421	(9.1)	385	(9.1)	36	(8.7)	

^1^ Pearson’s Chi-squared test.

**Table 2:** Comparison of sex and allergic factors between the MCS high-risk group and control group.

Comparative Items		Total Number n = 4630 (100%)		Control Group n = 4215 (91.0%)		High-Risk Group n = 415 (9.0%)		Crude Odds Ratio (95% CI ^3^)	*p*-Value ^1^
		n	(%)	N	(%)	n	(%)		
Sex	Male	2413	(52.1)	2238	(53.1)	175	(42.2)	1.00	<0.001
	Female	2217	(47.9)	1977	(46.9)	240	(57.8)	1.55	
								(1.27–1.90)	
Health condition									
SHS diagnosis	No	4611	(99.6)	4201	(99.7)	410	(98.8)	1.00	0.023
	Yes	19	(0.4)	14	(0.3)	5	(1.2)	3.67	
								(1.31–10.21)	
MCS diagnosis	No	4606	(99.5)	4195	(99.5)	411	(99.0)		0.267
	Yes	24	(0.5)	20	(0.5)	4	(1.0)		
Allergies ^2^	No	1023	(22.1)	950	(22.5)	73	(17.6)	1.00	0.022
	Yes	3607	(77.9)	3265	(77.5)	342	(82.4)	1.36	
								(1.05–1.77)	
Hay fever	No	1799	(38.9)	1656	(39.3)	143	(34.5)		0.054
	Yes	2831	(61.1)	2559	(60.7)	272	(65.5)		
Atopic dermatitis	No	3788	(81.8)	3468	(82.3)	320	(77.1)	1.00	0.009
	Yes	842	(18.2)	747	(17.7)	95	(22.9)	1.38	
								(1.08–1.76)	
Asthma	No	3852	(83.2)	3513	(83.3)	339	(81.7)		0.389
	Yes	778	(16.8)	702	(16.7)	76	(18.3)		
Mold/dust/mite	No	3379	(73.0)	3100	(73.5)	279	(67.2)	1.00	0.006
allergies	Yes	1251	(27.0)	1115	(26.5)	136	(32.8)	1.36	
								(1.09–1.68)	
Urticaria	No	3198	(69.1)	2934	(69.6)	264	(63.6)	1.00	0.012
	Yes	1432	(30.9)	1281	(30.4)	151	(36.4)	1.31	
								(1.06–1.62)	
Food allergies	No	3991	(86.2)	3653	(86.7)	338	(81.4)	1.00	0.003
	Yes	639	(13.8)	562	(13.3)	77	(18.6)	1.48	
								(1.14–1.93)	
Drug allergies	No	4503	(97.3)	4108	(97.5)	395	(95.2)	1.00	0.007
	Yes	127	(2.7)	107	(2.5)	20	(4.8)	1.94	
								(1.19–3.17)	
Metal allergies	No	4513	(97.5)	4121	(97.8)	392	(94.5)	1.00	<0.001
	Yes	117	(2.5)	94	(2.2)	23	(5.5)	2.57	
								(1.61–4.11)	

^1^ Pearson’s Chi-squared test or Fisher’s exact test. ^2^ Having one of the following allergies, namely hay fever, atopic dermatitis, asthma, mold/dust/mite allergies, urticaria, food allergies, drug allergies, and metal allergies, was defined as Yes. ^3^ CI, confidence interval.

**Table 3:** Comparison of the numbers of allergy-like symptoms between groups.

Comparative Items	Total Number n = 4630 (100%)			Control Group n = 4215 (91.0%)			High-Risk Group n = 415 (9.0%)			*p*-Value
	n (%)			n (%)			n (%)			
Count or allergy-like symptoms ^1^				Median value 1.0 [1.0–3.0]			Median value 2.0 [1.0–3.0]			<0.001 ^2^
					1.7 ± 1.5			2.1 ± 1.6		
No allergies	1023	(22.1)		950	(22.5)		73	(17.6)		
Has allergies	3607	(77.9)		3265	(77.5)		342	(82.4)		
One allergy-like symptom		1351	(29.2)		1242	(29.5)		109	(26.3)	
Two allergy-like symptoms		1029	(22.2)		936	(22.2)		93	(22.4)	
Three allergy-like symptoms		637	(13.8)		577	(13.7)		60	(14.5)	<0.001 ^3^
Four allergy-like symptoms		349	(7.5)		305	(7.2)		44	(10.6)	
Five allergy-like symptoms		165	(3.6)		144	(3.4)		21	(5.1)	
Six allergy-like symptoms		60	(1.3)		49	(1.2)		11	(2.7)	
Seven allergy-like symptoms		12	(0.3)		8	(0.2)		4	(1.0)	
Eight allergy-like symptoms		4	(0.1)		4	(0.1)		0	(0.0)	

^1^ We counted the number of allergy-like symptoms, including hay fever, atopic dermatitis, asthma, mold/dust/mite allergies, urticaria, food allergies, drug allergies, and metal allergies. If all were applicable, this counted as eight allergy-like symptoms. ^2^ Mann–Whitney U Test. ^3^ Pearson’s Chi-squared test.

**Table 4:** Comparison of residential environmental factors between the groups.

Comparative Items		Total Number n = 4630 (100%)		Control Group n = 4215 (91.0%)		High-Risk Group n = 415 (9.0%)		(95% CI ^2^) Crude Odds Ratio	*p*-Value ^1^
		n	(%)	n	(%)	n	(%)		
Number of times the respondent has experienced moving into a new home								(1.01–1.53)	0.036
Zero times		2157	(46.6)	1984	(47.1)	173	(41.7)	1.00	
One time or more		2473	(53.4)	2231	(52.9)	242	(58.3)	1.24	
Have you experienced home renovations, extensions, or redecorating?								(1.06–1.68)	0.015
	No	3642	(78.7)	3335	(79.1)	307	(74.0)	1.00	
	Yes	988	(21.3)	880	(20.9)	108	(26.0)	1.33	
Indoor environment									
Mold/condensation		1906	(41.2)	1723	(40.9)	183	(44.1)		0.204
Tatami room		4087	(88.3)	3713	(88.1)	374	(90.1)		0.220
Is there a freeway or national highway near your house?								(1.01–1.53)	0.044
	No	3078	(66.5)	2821	(66.9)	257	(61.9)	1.00	
	Yes	1552	(33.5)	1394	(33.1)	158	(38.1)	1.25	
Are there high-voltage power lines near your house?									0.839
	No	3815	(82.4)	3475	(82.4)	340	(81.9)		
	Yes	815	(17.6)	740	(17.6)	75	(18.1)		
Is there an intersection near your house?									0.196
	No	2061	(44.5)	1889	(44.8)	172	(41.4)		
	Yes	2569	(55.5)	2326	(55.2)	243	(58.6)		
Is there a railway near your house?									0.570
	No	3661	(79.1)	3328	(79.0)	333	(80.2)		
	Yes	969	(20.9)	887	(21.0)	82	(19.8)		
Is there a factory near your house?								(1.01–1.05)	0.045
	No	3544	(76.5)	3243	(76.9)	301	(72.5)	1.00	
	Yes	1086	(23.5)	972	(23.1)	114	(27.5)	1.26	
Is there a park near your house?									0.135
	No	2174	(47.0)	1994	(47.3)	180	(43.4)		
	Yes	2456	(53.0)	2221	(52.7)	235	(56.6)		
Is there a cropping field or orchard near your house?									0.401
	No	1853	(40.0)	1695	(40.2)	158	(38.1)		
	Yes	2777	(60.0)	2520	(59.8)	257	(61.9)		
Is there a golf course near your house?									0.179
	No	4272	(92.3)	3882	(92.1)	390	(94.0)		
	Yes	358	(7.7)	333	(7.9)	25	(6.0)		
Is there a gas station near your house?									0.112
	No	3444	(74.4)	3149	(74.7)	295	(71.1)		
	Yes	1186	(25.6)	1066	(25.3)	120	(28.9)		
Is there a rubbish dump near your house?								(1.08–2.10)	0.017
	No	4270	(92.2)	3900	(92.5)	370	(89.2)	1.00	
	Yes	360	(7.8)	315	(7.5)	45	(10.8)	1.51	
Do you smell offensive odors near your house?								(1.54–2.37)	<0.001
	No	3594	(77.6)	3320	(78.8)	274	(66.0)	1.00	
	Yes	1036	(22.4)	895	(21.2)	141	(34.0)	1.91	

^1^ Pearson’s Chi-squared test. ^2^ CI, confidence interval.

**Table 5:** Comparison of residential lifestyle between the groups.

Comparative Items		Total Number n = 4630 (100%)		Control Group n = 4215 (91.0%)		High-Risk Group n = 415 (9.0%)		Crude Odds Ratio (95% CI ^2^)	*p*-Value ^1^
		n	(%)	n	(%)	n	(%)		
Use of ventilation, exhaust fans									0.610
Yes		4081	(88.1)	3712	(88.1)	369	(88.9)		
No		549	(11.9)	503	(11.9)	46	(11.1)		
Use of insect repellant									0.335
	No	2780	(60.0)	2540	(60.3)	240	(57.8)		
	Yes	1850	(40.0)	1675	(39.7)	175	(42.2)		
Use of air fresheners									0.250
	No	1088	(23.5)	981	(23.3)	107	(25.8)		
	Yes	3542	(76.5)	3234	(76.7)	308	(74.2)		
Use of insecticides, electric mosquito coil									0.099
	No	1928	(41.6)	1771	(42.0)	157	(37.8)		
	Yes	2702	(58.4)	2444	(58.0)	258	(62.2)		
Use of floor wax									0.780
	No	3030	(65.4)	2761	(65.5)	269	(64.8)		
	Yes	1600	(34.6)	1454	(34.5)	146	(35.2)		
Extermination of termites and mites									0.176
	No	3784	(81.7)	3455	(82.0)	329	(79.3)		
	Yes	846	(18.3)	760	(18.0)	86	(20.7)		
Use of agricultural chemicals, herbicides									0.850
	No	3564	(77.0)	3243	(76.9)	321	(77.3)		
	Yes	1066	(23.0)	972	(23.1)	94	(22.7)		
Use of a kerosene stove									0.151
	No	2242	(48.4)	2055	(48.8)	187	(45.1)		
	Yes	2388	(51.6)	2160	(51.2)	228	(54.9)		
Use of an air purifier									0.708
	Yes	1868	(40.3)	1697	(40.3)	171	(41.2)		
	No	2762	(59.7)	2518	(59.7)	244	(58.8)		
Has pets									0.379
	No	2538	(54.8)	2302	(54.6)	236	(56.9)		
	Yes	2092	(45.2)	1913	(45.4)	179	(43.1)		

^1^ Pearson’s Chi-squared test. ^2^ CI, confidence interval.

**Table 6:** Comparison of lifestyle factors between the groups.

Comparative Items		Total Number n = 4630 (100%)		Control Group n = 4215 (91.0%)		High-Risk Group n = 415 (9.0%)		Crude Odds Ratio (95% CI ^2^)	*p*-Value ^1^
		n	(%)	n	(%)	n	(%)		
Eats breakfast every day									0.483
Yes		4184	(90.4)	3813	(90.5)	371	(89.4)		
No		446	(9.6)	402	(9.5)	44	(10.6)		
Considers a balanced nutritional intake									0.095
	Yes	2412	(52.1)	2212	(52.5)	200	(48.2)		
	No	2218	(47.9)	2003	(47.5)	215	(51.8)		
Frequent consumption of instant foods									0.882
	No	2147	(46.4)	1956	(46.4)	191	(46.0)		
	Yes	2483	(53.6)	2259	(53.6)	224	(54.0)		
Meals are mainly Western cooking									0.627
	No	2896	(62.5)	2641	(62.7)	255	(61.4)		
	Yes	1734	(37.5)	1574	(37.3)	160	(38.6)		
Frequent consumption of fermented foods such as miso and pickles									0.094
	Yes	3294	(71.1)	2984	(70.8)	310	(74.7)		
	No	1336	(28.9)	1231	(29.2)	105	(25.3)		
Frequent consumption of vegetables									0.300
	Yes	3427	(74.0)	3111	(73.8)	316	(76.1)		
	No	1203	(26.0)	1104	(26.2)	99	(23.9)		
Drinking water is from a water purifier or is bought water									0.147
	Yes	1876	(40.5)	1694	(40.2)	182	(43.9)		
	No	2754	(59.5)	2521	(59.8)	233	(56.1)		
Frequent consumption of green tea from plastic bottles (made from polyethylene terephthalate)									0.944
	No	2105	(45.5)	1917	(45.5)	188	(45.3)		
	Yes	2525	(54.5)	2298	(54.5)	227	(54.7)		
Exercises more than once a week (outside of physical education class)									0.006
	Yes	2875	(62.1)	2643	(62.7)	232	(55.9)	1.00	
	No	1755	(37.9)	1572	(37.3)	183	(44.1)	1.33	
								(1.08–1.63)	
Soaks in the bathtub when bathing									0.712
	Yes	2817	(60.8)	2561	(60.8)	256	(61.7)		
	No	1813	(39.2)	1654	(39.2)	159	(38.3)		
Usage time of PC/smartphone									0.181
Less than 3 h		2209	(47.7)	2024	(48.0)	185	(44.6)		
3 h or more		2421	(52.3)	2191	(52.0)	230	(55.4)		
Television viewing time									0.470
Less than 2 h		2220	(47.9)	2014	(47.8)	206	(49.6)		
2 h or more		2410	(52.1)	2201	(52.2)	209	(50.4)		

^1^ Pearson’s Chi-squared test. ^2^ CI, confidence interval.

**Table 7:** Comparison of lifestyle habits and physical/mental conditions between the groups.

Comparative Items		Total Number n = 4630 (100%)		Control Group n = 4215 (91.0%)		High-Risk Group n = 415 (9.0%)		Crude Odds Ratio (95% CI ^2^)	*p*-Value ^1^
		n	(%)	N	(%)	n	(%)		
Smoking habit 1									0.110
Non-smoker		4500	(97.2)	4097	(97.2)	403	(97.1)		
Former smoker		119	(2.6)	110	(2.6)	9	(2.2)		
Smoker		11	(0.2)	8	(0.2)	3	(0.7)		
Smoking habit 2									0.069
Non-smoker or former smoker		4619	(99.8)	4207	(99.8)	412	(99.3)		
Smoker		11	(0.2)	8	(0.2)	3	(0.7)		
Family member who smokes									0.213
	No	1726	(37.3)	1583	(37.6)	143	(34.5)		
	Yes	2904	(62.7)	2632	(62.4)	272	(65.5)		
Drinking habit									0.176
Does not drink alcohol		4481	(96.8)	4084	(96.9)	397	(95.7)		
Drinks alcohol sometimes or every day		149	(3.2)	131	(3.1)	18	(4.3)		
Cold hands and feet									<0.001
	No	2910	(62.9)	2702	(64.1)	208	(50.1)	1.00	
	Yes	1720	(37.1)	1513	(35.9)	207	(49.9)	1.78	
								(1.45–2.18)	
Fatigue									<0.001
	No	2250	(48.6)	2111	(50.1)	139	(33.5)	1.00	
	Yes	2380	(51.4)	2104	(49.9)	276	(66.5)	1.99	
								(1.61–2.46)	
Hours of sleep									0.064
7–8 h		2298	(49.6)	2110	(50.1)	188	(45.3)		
9 h or more or 6 h or less		2332	(50.4)	2105	(49.9)	227	(54.7)		
Bed time									0.138
Before 11 p.m.		431	(9.3)	384	(9.1)	47	(11.3)		
After 11 p.m.		4199	(90.7)	3831	(90.9)	368	(88.7)		
Normal temperature									0.148
34.5–35.9 °C		1450	(31.3)	1307	(31.0)	143	(34.5)		
36.0 °C or higher		3180	(68.7)	2908	(69.0)	272	(65.5)		
Subjective stress 1									<0.001
Slight		1307	(28.2)	1247	(29.6)	60	(14.5)	1.00	
Moderate–significant		3323	(71.8)	2968	(70.4)	355	(85.5)	2.49	
								(1.88–3.29)	
Subjective stress 2									<0.001
Slight–moderate		3826	(82.6)	3528	(83.7)	298	(71.8)	1.00	
Significant		804	(17.4)	687	(16.3)	117	(28.2)	2.02	
								(1.60–2.54)	

^1^ Pearson’s Chi-squared test. ^2^ CI, confidence interval.

**Table 8:** Inter-group comparison of the three QEESI category scores.

QEESI ^2^		Control Group n = 4215 (91.0%)	High-Risk Group n = 415 (9.0%)	Difference	*p*-Value ^1^
Q1 Chemical Intolerance	Mean ± standard deviation	16.1 ± 16.0	51.7 ± 8.5	35.6	
	Median (25–75% points)	12 (2–26)	50 (46–56)	38.0	<0.001
	Minimum to maximum	0–100	40–100	-	
Q3 Symptom Severity	Mean ± standard deviation	18.6 ± 15.3	45.5 ± 13.6	26.9	
	Median (25–75% points)	15 (6–28)	46 (35–54)	31.0	<0.001
	Minimum to maximum	0–84	20–93	-	
Q5 Life Impact	Mean ± standard deviation	5.5 ± 7.3	25.9 ± 14.3	20.4	
	Median (25–75% points)	3 (0–8)	22 (14–34)	19.0	<0.001
	Minimum to maximum	0–63	10–100	-	

^1^ Mann–Whitney U Test. ^2^ QEESI, Quick Environment Exposure Sensitivity Inventory.

**Table 9:** Factors related to the MCS high-risk group based on logistic regression analysis.

Variables	Univariate Analysis		*p*-Value ^1^	Multivariable Analysis		*p*-Value ^1^
	Crude Odds Ratio	95% CI ^3^		Adjusted Odds Ratio	95% CI ^3^	
Sex, female [male] ^2^	1.55	1.27–1.90	<0.001	1.29	1.04–1.60	0.020
Atopic dermatitis [no]	1.38	1.08–1.76	0.009	1.29	1.01–1.65	0.045
Metal allergies [no]	2.57	1.61–4.11	<0.001	1.79	1.11–2.91	0.018
Cold hands and feet [no]	1.78	1.45–2.18	<0.001	1.36	1.09–1.69	0.006
Number of times moving to a new home/one time or more [0 times]	1.24	1.01–1.53	0.036	1.26	1.02–1.55	0.030
Offensive odor [no]	1.91	1.54–2.37	<0.001	1.69	1.35–2.10	<0.001
Bedtime [after 11 p.m.]	0.79	1.57–1.08	0.138	1.40	1.01–1.95	0.044
Subjective stress 1/moderate–significant [slight]	2.49	1.88–3.29	<0.001	2.02	1.51–2.71	<0.001
Fatigue [no]	1.99	1.61–2.46	<0.001	1.45	1.15–1.82	0.002
The Hosmer–Lemeshow test resulted in *p* = 0.976 and the model’s discriminant accuracy was 91.0%						

^1^ Pearson’s Chi-squared test. ^2^ The reference categories are specified in square brackets. ^3^ CI, confidence interval.

## Data Availability

The datasets used and/or analyzed during the current study are available from the corresponding author on reasonable request.
